# GLP-1 Receptor Agonists: A Promising Therapy for Modern Lifestyle Diseases with Unforeseen Challenges

**DOI:** 10.3390/ph17111470

**Published:** 2024-11-01

**Authors:** Patrycja Kupnicka, Małgorzata Król, Justyna Żychowska, Ryszard Łagowski, Eryk Prajwos, Anna Surówka, Dariusz Chlubek

**Affiliations:** 1 Department of Biochemistry and Medical Chemistry, Pomeranian Medical University in Szczecin, Powstańców Wlkp. 72, 70-111 Szczecin, Poland; 2 Department of Plastic, Endocrine and General Surgery, Pomeranian Medical University, 72-010 Szczecin, Poland

**Keywords:** glucagon-like peptide-1, GLP-1 receptor agonists, obesity, cancer, inflammation

## Abstract

Modern lifestyle diseases remain a persistent challenge in healthcare. Currently, about 422 million people worldwide are affected by diabetes, while 1 in 8 people are living with obesity. The development of glucagon-like peptide 1 receptor agonists (GLP-1RAs) has marked a significant milestone in treating these conditions. Interest in GLP-1RAs has grown due to evidence that, beyond their established role in diabetes management, these drugs influence other metabolic disorders. This is attributed to the fact that GLP-1 receptors are found in various healthy human tissues. However, a potential cause for concern is the expression of GLP-1 receptors in certain cancers. This review focuses on the most recent findings concerning the actions of GLP-1RAs, detailing their documented impact on the thyroid gland and pancreas. It addresses concerns about the long-term use of GLP-1RAs in relation to the development of pancreatitis, pancreatic cancer, and thyroid neoplasms by exploring the mechanisms and long-term effects in different patient subgroups and including data not discussed previously. This review was conducted through an examination of the literature available in the MedLine (PubMed) database, covering publications from 1978 to 10 May 2024. The collected articles were selected based on their relevance to studies of GLP-1 agonists and their effects on the pancreas and thyroid and assessed to meet the established inclusion criteria. The revised papers suggest that prolonged use of GLP-1RA could contribute to the formation of thyroid tumors and may increase the risk of acute inflammatory conditions such as pancreatitis, particularly in high-risk patients. Therefore, physicians should advise patients on the need for more frequent and detailed follow-ups.

## 1. Introduction

Modern lifestyle diseases remain a persistent challenge in healthcare. The most common are diabetes, obesity, and cardiovascular diseases. About 422 million people worldwide have diabetes, and 1 in 8 people in the world are living with obesity. Recently, agonists of the glucagon-like peptide 1 receptor (GLP-1RA) have garnered particular interest within both the medical and scientific communities. The sudden surge in popularity and substantial attention toward these anti-diabetic medications can be attributed to their application for weight reduction, even among individuals without type 2 diabetes [1,2]. Their action is analogous to the action of endogenous GLP-1 and primarily involves regulating the carbohydrate metabolism in the human body, promoting satiety, and reducing food desire [3], leading to an improvement in insulin signaling metabolism and weight loss. However, despite the growing interest in therapy utilizing GLP-1RA, it is important to bear in mind that they are relatively novel medications and not all the long-term effects of their usage have been elucidated.

### 1.1. The Structure, Synthesis, Action, and Catabolism of GLP-1

Glucagon-like peptide-1 is a peptide hormone composed of 30–31 amino acids that are produced and secreted in the endocrine L cells of the intestinal epithelium, as well as certain neurons within the nucleus of the solitary tract, in response to ingested meals. It is generated through tissue-specific post-translational modifications of the proglucagon molecule. The expression of the proglucagon gene increases in the pancreas during fasting but decreases (and is stimulated in the postprandial state) in the brain and intestines [4]. Within the gut and brain, proglucagon is cleaved by prohormone convertase 1/3 (PC 1/3), leading to the formation of glicentin, which can be further processed into glicentin-related pancreatic polypeptide (GRPP) and oxyntomodulin, GLP-1, intervening peptide-2 (IP-2), and glucagon-like peptide-2 (GLP-2). Initially, the full-length GLP-1 (1–37) is enzymatically catalyzed into the biologically active GLP-1 (7–37). Subsequently, the glycine at position 108 in proglucagon serves as a substrate for amidation of the C-terminal arginine, resulting in the equally potent GLP-1 (7–36) amide. In humans, over 80% of secreted GLP-1 is amidated, whereas a significant portion remains as GLP-1 (7–37) in other species [4].

GLP-1 belongs to the incretin family, which, among other functions, increases post-meal insulin secretion by pancreatic β-cells, influences enterogastrones that control gastric motility, and decreases postprandial glucagon release [5,6], thereby preventing post-meal glucose fluctuations [4,7,8]. GLP-1′s action also encompasses appetite control through its interaction with specific receptors in the brain [8]. The peptide enhances muscle insulin sensitivity, promotes lipolysis, and reduces lipogenesis [9]. The incretin effect induced by GLP-1 is initiated in the gastrointestinal tract following the consumption of meals containing glucose, lipids, amino acids, or mixed meals [4,10]. The activity of this hormone is rapidly attenuated (with a half-life of approximately 2 min) by the enzymes dipeptidyl peptidase-4 (DPP-4) and neutral endopeptidase [4,8,11,12].

### 1.2. GLP-1 Receptors

GLP-1 receptors are encoded by the GLP-1R gene located on chromosome 6 in humans [13]. Each receptor is divided into an extracellular domain that binds to GLP-1 and a transmembrane domain [14]. They belong to the group of G protein-coupled receptors, and their activation leads to an increase in intracellular cAMP levels [15].

In humans, GLP-1 receptors are found in the pancreas, gastrointestinal tract, heart, lungs, kidneys, brain, and adipose tissue [16,17,18]. However, their expression has not been demonstrated in lymph nodes, spleen, and liver [19], although their expression has been confirmed in thyroid C cells [20,21]. The presence of specific GLP-1 receptors in the brain is likely responsible for the prolonged feeling of satiety after administration of analogs of this hormone [22], and it regulates the secretion of ACTH, aldosterone, and corticosterone by influencing the hypothalamic–pituitary–adrenal axis [23,24].

**Figure 1:**
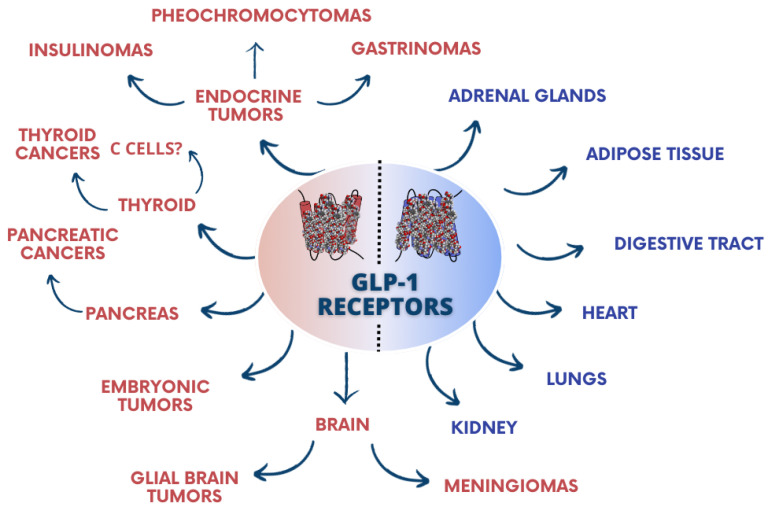
Expression of GLP-1 receptors in healthy (blue) and tumor (red) tissues in human subjects.

Moreover, the presence of GLP-1 receptors in humans has also been identified in endocrine tumors, particularly insulinomas, gastrinomas, and pheochromocytomas, as well as in glial brain tumors, meningiomas, and embryonic tumors. However, these receptors are nearly absent in carcinomas and lymphomas [19] (Figure 1).

### 1.3. GLP-1RA and Receptor Agonists and Their Application

GLP-1RAs constitute a relatively new group of drugs, synthetic counterparts of human GLP-1, typically administered through subcutaneous injections [25,26]. Based on their duration of action, they can be classified into short-acting, including exenatide, lixisenatide, and beinaglutide, and long-acting, such as liraglutide, dulaglutide, albiglutide, semaglutide, PEG-loxenatide, and tirzepatide [27] (Table 1).

**Figure 2:**
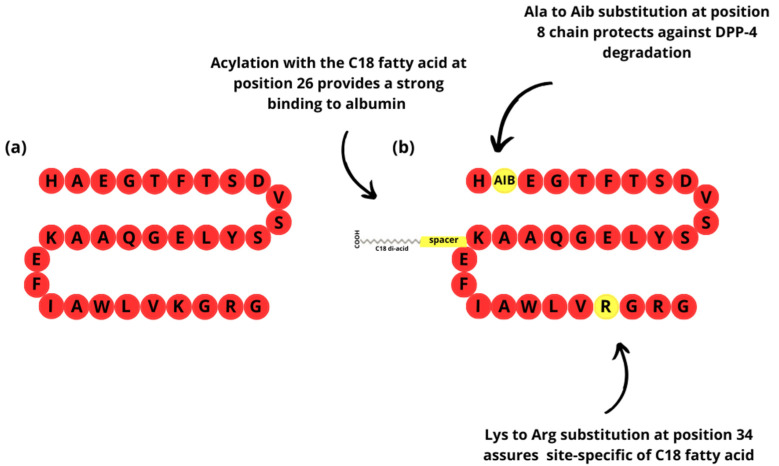
The structure of the human endogenous GLP-1 (fragment 7–37) (**a**) and GLP-1 analog—semaglutide (subcutaneous formulation) (**b**). The modifications of the polypeptide chain of GLP-1 analog provide additional properties to the molecule, such as longer half-life (stronger binding to albumin and prevention against degradation). That allows the drug to take action in the system longer than endogenous GLP-1 and its administration every few days. Aib-2—aminoisobutyric acid; DPP-4—dipeptidyl peptidase-4.

Their amino acid sequences are either identical or highly similar to natural GLP-1. The molecules themselves have undergone certain modifications, such as the addition of fatty acid side chains, polyethylene glycol (PEG) modification, or Fc-fusion, to achieve greater stability and/or activity of the molecule [28] (Figure 2).

GLP-1RA are primarily utilized for the treatment of type 2 diabetes [3], and they also show potential in combating obesity [29,30,31]. Their binding to GLP-1 receptors induces the same effects as the binding of their natural agonist (increased insulin secretion, decreased glucagon secretion, anorexigenic effect), which, over the long term, leads to changes in cellular metabolism at the level of gene expression and translation [32]. Tissue sensitivity to insulin increases, while the expression of genes related to fatty acid synthesis in the liver is downregulated, limiting fat accumulation in the liver [33]. The synthesis of stearoyl-CoA desaturase (SCD), which is associated with alcohol-related liver disease, is also decreased by GLP-1RA [34]. Furthermore, a multicenter, double-blind, randomized, placebo-controlled phase 2 study in humans demonstrated that treatment with liraglutide was safe, well-tolerated, and led to the histological resolution of non-alcoholic steatohepatitis [35]. Consequently, GLP-1RA treatment may prevent non-alcoholic fatty liver and prove beneficial in treating existing disease [35,36].

Studies suggest that GLP-1RA may have applications in treating alcohol and drug addiction [37,38], including opioids [39] and cigarette smoking [40]. Some research also indicates a favorable impact of GLP-1RA therapy on fertility in women with polycystic ovary syndrome [41]. However, official information in the drug leaflets of GLP-1RA prohibits their use during pregnancy due to their adverse effects on pregnancy development in animals [42] and limited knowledge about their use during pregnancy in women with diabetes [43].

The wide spectrum of effects elicited by natural GLP-1 and its agonists has led to significant interest among researchers. The metabolic changes induced by these drugs and the presence of their receptors in various tissues extend their influence beyond pancreas-related insulin signaling and blood glucose regulation, potentially modulating endocrine activity in other glandular tissues. Additionally, alongside the positive outcomes of GLP-1RA therapy, there is an increasing body of scientific reports discussing potential negative consequences of these drugs, such as an increased risk of cancer [20,44,45].

## 2. Methods

This systematic review was undertaken according to the PRISMA reporting guidelines [46]. The literature searches of free text and MeSH terms were performed using MedLine (PubMed) from 1978 to 10 May 2024. All of the searches were performed using a combination of subject headings and free-text terms. The final search strategy was determined through several pre-searches. The keywords used in the search strategy were “glp-1 and pancreas” OR “glp-1 and thyroid”.

### Eligibility Criteria

**Figure 3:**
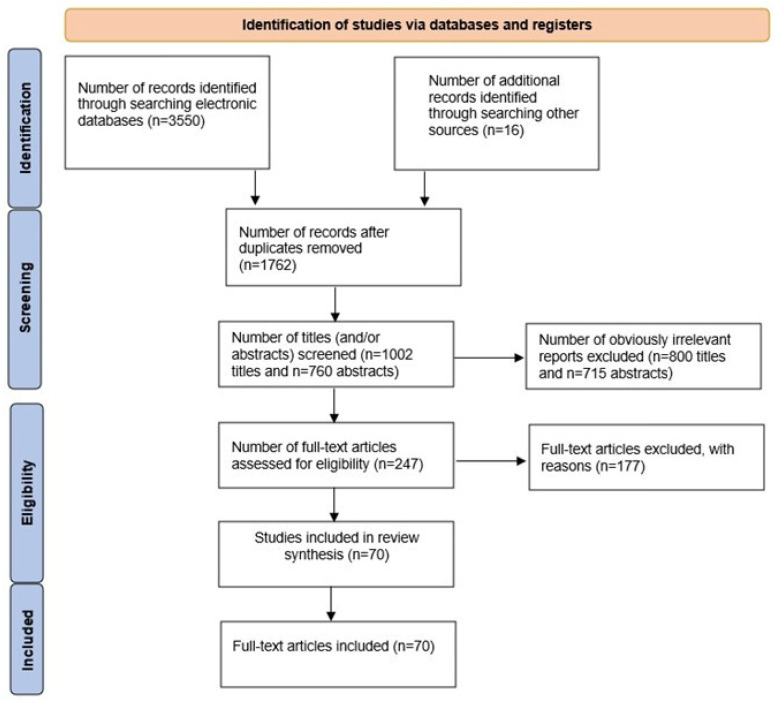
Summary of study search and selection.

The following inclusion criteria were employed for this review: (1) prospective clinical trials; (2) retrospective clinical trials; (3) in vivo and in vitro studies; (4) studies published in English. All potentially assessed articles aimed to investigate the GLP-1 receptor agonists and their effects on the thyroid and pancreas to update and evaluate the accuracy and reliability of the existing reports. Studies were deemed eligible if the following criteria were met: (a) animals or patients were treated with GLP-1 RA; (b) long-acting interventions, short-acting GLP-1 RAs were used; (c) the studies compared intervention(s) with placebo or other diabetes drugs; (d) outcomes considered pancreatic or thyroid complications including thyroid hormones, and TSH changes, thyroid cancer, changes in pancreatic enzymes levels, pancreatitis, and pancreatic cancer. The following exclusion criteria were applied: (1) lack of statistical analysis; (2) papers related to GLP-1 but not GLP-1 receptor agonists; (3) papers related to aspects of the GLP-1 treatment other than pancreatic or thyroid disorders; and (4) the unreviewed literature (Figure 3). All duplicates were removed by exporting the retrieved articles to Zotero software version: 6.0.36 (Corporation for Digital Scholarship, Vienna, VA, USA). Two independent researchers (P.K. and M.K.) evaluated the remaining articles by reviewing the title and abstract. The full text was then assessed to ensure the inclusion of relevant articles. Any disagreements were resolved by consensus or discussion with another investigator (J.Ż).

## 3. GLP-1RA Influence on Thyroid Function

Numerous available drugs can interact with thyroid function, even those not specifically prescribed to modulate gland activity. These include antibiotics such as minocycline and tetracycline, cardiac medications like amiodarone, psychotropic drugs (lithium), and immunosuppressive drugs (pembrolizumab) [47]. However, research has also demonstrated that anti-diabetic medications like GLP-1RA (such as liraglutide, semaglutide, and dulaglutide) might impact thyroid gland metabolism, potentially leading to the development of thyroid cancer [47,48,49].

### 3.1. The Influence on TSH Levels

There are reports that exenatide, registered as a medication for use in type 2 diabetes [50], decreases serum TSH levels in patients who have reduced their body weight. In a study by Su Ann Tee et al., TSH levels were assessed in 112 patients before and after 12 months of exenatide therapy. As expected, this study confirmed a significant decrease in serum TSH levels in patients, particularly those with additional weight loss [51]. However, the concentration of free thyroxine (FT4) remained unchanged. Since TSH is produced by hypothalamic neurons, the obtained results may suggest a connection between the observed effects and the expression of GLP-1 receptors in the central nervous system [51,52]. The decrease in TSH levels might also be associated with weight loss alone, as increased body weight tends to elevate TSH levels [53], implying that the reverse situation might lead to contrasting effects [54,55,56].

The absence of changes in FT4 concentration could also be due to increased tissue sensitivity to thyroid hormones [56]. Köseoğlu et al. conducted a study involving 39 patients with type 2 diabetes who were administered exenatide for 6 months. After this period, a reduced thyroid volume and decreased TSH levels were observed in these patients. The levels of hormones FT3, FT4, and calcitonin did not change. Thyroid nodules were also evaluated, and significant differences were not noted in their morphology during exenatide use [57].

Similar results were obtained in another study involving 46 diabetes patients without thyroid disorders. After a 6-month exenatide treatment, no increase in the thyroid volume was observed [56]. However, a significant reduction in serum TSH levels was seen, along with normal FT3 and FT4 levels. Furthermore, there was no increase in anti-thyroid antibodies, which contradicts the notion of thyroid autoimmunization following exenatide use [56,58].

Studies also describe the benefits of using liraglutide in patients with non-alcoholic fatty liver disease (NAFLD). Thyroid hormones (TH) play a role in liver lipid metabolism, making elevated TSH and reduced FT4 levels risk factors for NAFLD [59,60,61]. Resistance in the TH pathway within the liver leads to lipid accumulation, resulting in NAFLD [62]. A significant discovery was the indication that liraglutide can lower TSH levels in patients independently of their liver condition, leading to reduced liver resistance to thyroid hormones [61].

### 3.2. C-Cell Hyperplasia and Thyroid Cancer

#### GLP-1RA and Safety of Use

In the latest studies involving 2562 patients, it has been demonstrated that prolonged use, specifically for 1–3 years, of GLP-1 receptor agonists is associated with an increased risk of various types of thyroid tumors [44].

The European Medicines Agency (EMA) has provided access to all the reports registered in EudraVigilance related to GLP-1RA: exenatide; liraglutide; lixisenatide; albiglutide; dulaglutide; and semaglutide, from the introduction of each GLP-1RA up to 30 January 2020. These reports include terms such as “medullary thyroid carcinoma”, “thyroid adenoma”, “thyroid cancer”, and “thyroid cancer with metastasis”, where the medications were considered suspected or interacting [63]. A total of 6,665,794 adverse events were reported during the study period, including 11,243 cases of thyroid cancer. Among them, 236 patients were taking GLP-1RA. Liraglutide, dulaglutide, and exenatide met the criteria for generating a safety signal, suggesting that thyroid cancer occurs as an adverse event more frequently than with other drugs. The strongest association was observed with liraglutide, followed by exenatide [63]. Ultimately, the results from the EudraVigilance database do not confirm a relationship between C-cell hyperplasia and the use of GLP-1RA.

In the report generated by Yang et al. (2022), which considers the data from the FDA Adverse Event Reporting System (FAERS) collected between the first quarter of 2004 and the second quarter of 2011, 8718 reported cases of cancer were diagnosed in patients using GLP-1RA. In the reports on the thyroid, malignant tumors predominated (698 cases) over benign tumors (85 cases), and the dominant malignant tumor was papillary carcinoma, accounting for 28.9% of cases, followed by medullary carcinoma (11.0% of cases). As the authors themselves point out, it is impossible to unequivocally assess the effect of GLP-1RA on the risk of thyroid cancer due to other factors. The increased number of tumors could have been influenced by factors such as patients taking other medications (metformin, levothyroxine) or the constantly growing number of screening tests for these tumors in recent years [64]. Therefore, the relationship between GLP-1RA and thyroid cancers requires continuous monitoring of the effects of these medications on the thyroid. Also, a more comprehensive diagnostic approach to detecting pathological changes in the thyroid is crucial. Safety warnings necessitate expanding and prolonging long-term studies to detect potential risks, especially when using liraglutide, dulaglutide, and exenatide [63,64].

### 3.3. C-Cell Hyperplasia and Medullary Thyroid Carcinoma

Medullary thyroid carcinoma is a tumor originating from the calcitonin-producing neuroendocrine C cells of the thyroid. This type of cancer constitutes only 0.6% of all thyroid cancers in Korea and 1% to 2% in the United States [65]. It is commonly associated with multiple endocrine neoplasia (MEN) type 2, a genetic disorder characterized by the presence of tumors in the thyroid, parathyroid, and adrenal glands [66], caused by activation of a cellular oncogene, RET [67]. However, the incidence of this cancer may be related to the use of GLP-1RA, such as semaglutide, liraglutide, dulaglutide, or exenatide [68].

It has been demonstrated that liraglutide activated GLP-1 receptors on thyroid C cells in rodents [20]. Stimulation of GLP-1RA by commercial analogs leads to an increase in the expression of the calcitonin gene, which, due to its high specificity and sensitivity, is considered a clinical biomarker for thyroid diseases originating from C cells [69]. This enhanced hormone synthesis subsequently leads to hyperplasia of C cells in both male and female mice and rats, which could evolve into medullary thyroid carcinoma [20,48,70,71,72,73].

Similar effects of therapy have been observed with semaglutide and exenatide, where drug administration resulted in thyroid C cell hyperplasia, increasing the risk of developing adenomas and cancers originating from these cells. Interestingly, the same study reported no negative impact of GLP-1RA on parathyroid gland morphology or serum calcium concentration [74].

There are interspecies differences in the number of thyroid C cells and GLP-1RA expression, as demonstrated by Knudsen et al. in studies involving rats, mice, cynomolgus monkeys, and human thyroid tissues [20]. The presence of GLP-1RA within the thyroid was unique to C cells, and the number of C cells in mice and rats was about 45 times higher than in humans and monkeys. This study showed minimal mRNA expression of the GLP-1 receptor in humans, no expression of its protein in thyroid C cells, and no significant increase in calcitonin levels after liraglutide treatment (single and repeated doses). The experiment on rodents’ C cells exposed to liraglutide or exenatide demonstrated a significant increase in cAMP, contributing to an immediate rise in calcitonin concentration. However, Knudsen et al. did not observe an increase in cAMP levels in human thyroid C cells after administration of the GLP-1RA. Long-term high-dose liraglutide exposure led to C-cell hyperplasia and tumor (adenomas and carcinomas) formation in rodents; however, it did not influence nonhuman primates’ C-cells [20].

Studies in monkeys have not shown a correlation between the administration of GLP-1RA and C-cell hyperplasia [20]. Among monkeys treated with liraglutide at a dose 60 times higher than the recommended human dose, no abnormalities related to thyroid C cell function were observed even after 20 months of treatment, suggesting a minimal presence of GLP-1RA receptors in primates. Additionally, after two years of liraglutide use, there was no increase in calcitonin levels in the study group [20]. The lack of calcitonin increase from human C cells upon liraglutide use might indicate a different mechanism of C cell hyperplasia, not necessarily associated with C cell activation by calcitonin and thyroid cancer development [20,49].

Similar results were obtained in one of the first long-term studies (36 months) analyzing the effects of liraglutide on C cells, where the authors did not find a connection between liraglutide use and an increase in calcitonin levels or C cell hyperplasia [75]. Also, in their clinical studies involving over 5000 individuals with type 2 diabetes or obese individuals without diabetes, Hegedus et al. (2011) showed that with liraglutide and exenatide use over 2 years, calcitonin levels gradually increased over time but without clinical significance [76]. A case report by Zou et al. showed that continuing tirzepatide administration after thyroidectomy in a diabetic patient treated with GLP-1 RA with increased calcitonin level (140 pg/mL, reference value 0–7.5 pg/mL) and reactive C cell hyperplasia without germline mutation of RET gene, did not influence the level of calcitonin and did not show any abnormalities in the ultrasound exam (8 months follow-up) [77]. Nevertheless, the authors emphasize that monitoring thyroid function in patients receiving GLP-1 analogs/receptor agonists is recommended, especially in the presence of other conditions that may increase the risk of CCH/thyroid cancer [77].

The anatomical and physiological characteristics of primates and rodents are distinct from each other. Humans and primates have a lower density of C cells in the thyroid compared to rodents [20]. This might be due to the fact that in rodents, calcitonin plays a more significant role in calcium regulation [78,79]. Primates, including monkeys and humans, possess thyroid C cells that likely do not exhibit significant GLP-1 receptor expression compared to rodent C cells [80,81].

On the other hand, some studies confirm the expression of GLP-1 receptors in already existing medullary thyroid carcinoma and C-cell hyperplasia [19,21]. Song et al. (2017) demonstrated that 30 out of 59 patients with medullary thyroid carcinoma exhibited membrane receptor GLP-1 expression, accounting for 50.8% of the studied cases [82]. The authors also highlighted that the increase in GLP-1 receptor expression in patients with medullary thyroid carcinoma rises with age [74,82]. Other authors pointed out that the rare presence of GLP-1R-positive medullary thyroid carcinoma might be a candidate for in vivo scintigraphy and targeted radiotherapy [18]. An intriguing aspect is the potential outcome of treating medullary thyroid carcinoma with GLP-1 receptor antagonists, given that GLP-1 agonists might influence cancer development [83].

Medullary thyroid carcinoma is a relatively rare tumor, accounting for 3–5% of all thyroid cancers, and it leads to death in approximately 13% of patients [84,85,86]. The incidence increases with age [87]. Caution is warranted when using GLP-1RA in individuals with an elevated risk of medullary thyroid carcinoma.

Mutations in the RET proto-oncogene occur in about 25% of medullary thyroid carcinomas, leading to hereditary cancer syndromes such as MEN2A and MEN2B [88]. Patients at a heightened risk of developing medullary thyroid carcinoma originating from C-cells or multiple endocrine neoplasia type 2 (MEN2) who have a family history of the disease, according to information provided in the package inserts of GLP-1RA preparations in the USA, should not be treated with GLP-1RA [89,90]. Available data indicate that definitively excluding a connection between GLP-1RA and thyroid pathologies is challenging. Consequently, registries for annual incidence of medullary thyroid carcinoma have been established in the USA. This monitoring is expected to continue for approximately 15 years, and the results can be anticipated around 2035–2037 [84].

### 3.4. Papillary Thyroid Carcinoma

In another type of thyroid cancer, papillary thyroid carcinoma, Gier et al. demonstrated the expression of GLP-1 receptors [21]. In their study, GLP-1 receptor expression was found in 3 out of 17 papillary thyroid carcinoma cases, which should direct further research not only toward the rare medullary thyroid carcinoma but also the most common type of thyroid cancer, papillary thyroid carcinoma, constituting 90% of all thyroid cancers [91].

In the research conducted by Jung et al., immunohistochemical staining of 56 tissues from patients with papillary thyroid carcinoma indicated that 18 of them exhibited cytoplasmic GLP-1 receptor expression, while healthy thyroid tissues did not show such expression. Immunoreactivity was also present in two out of seven cases of nodular hyperplasia [92]. Similarly, among the Egyptian population of 80 healthy controls and 80 patients with papillary thyroid carcinoma (PTC), increased GLP-1 receptor expression was observed in PTC patients who were homozygous for the AA allele at the rs1042044 A locus, which is associated with the GLP-1 receptor polymorphism. Furthermore, a positive association was found with worse clinical status, such as tumor multifocality, lymph node metastases, and GLP-1 receptor expression [93].

The latest studies do not show an association between the use of GLP-1 receptor agonists and a substantially increased risk of thyroid cancer in the Scandinavian population over a mean follow-up of 3.9 years [94]. Also, the analysis conducted by Sungho Bea in 2023 suggests no differences in thyroid cancer risk in patients using GLP-1RA and/or DPP-4 inhibitors and sodium–glucose cotransporter-2 (SGLT2) inhibitors in patients with type 2 diabetes in South Korea [95].

## 4. The Impact of GLP-1RA on the Pancreas

The exocrine portion of the pancreas secretes digestive enzymes and bicarbonates, which are essential for the digestion and absorption of food, while the endocrine part produces and releases peptide hormones—insulin and glucagon—to regulate blood glucose levels [96]. The presence of GLP-1 receptors on the surface of pancreatic cells and the stimulation of insulin secretion by GLP-1 make this organ one of the key targets for GLP-1RA. GLP-1RAs have shown significant effectiveness in treating type 2 diabetes. However, some studies have associated the use of GLP-1RA with an increased risk of pancreatitis and pancreatic cancer [97,98], although the findings in this matter are not conclusive.

### 4.1. GLP-1RA and the Function of Beta Cells

GLP-1RAs, by interacting with specific receptors on pancreatic beta cells, stimulate the secretion of insulin [99]. There are reports that the activation of GLP-1 receptors induces an increase in cAMP concentration, which affects ATP-sensitive K+ and Ca2+ channels that stimulate insulin secretion. Additionally, mechanistically, GLP-1RA, by activating cAMP, affects the mTOR signaling pathway in β-cells, which stimulates HIF-1α. Consequently, higher expression of HIF-1α indicates transcription of glycolytic genes, which increases glucose-stimulated insulin secretion [100]. Zander et al. demonstrated that a six-week therapy with GLP-1RA improves the function of pancreatic beta cells [101]. In the LEAD-6 studies comparing the efficacy and safety of liraglutide with exenatide, it was shown that GLP-1 treatment led to an increase in fasting insulin. Moreover, the homeostasis model assessment index of β-cell function (HOMA-B) showed significantly greater improvement in the liraglutide group compared to the exenatide group, indicating more favorable effects of liraglutide on enhancing beta cell function [102]. Other drugs that enhance glucose-dependent insulin secretion are albiglutyd [103], lixisenatide [104], dulaglutide [105], and tirzepatide [106].

Individuals with type 2 diabetes often experience impaired insulin secretion in the first phase [107], the study by T. Vilsbøll confirmed that liraglutide increased insulin secretion in both the first phase (approximately 2 min after the rise in blood glucose levels) and the second phase [108]. Significantly increased first-phase insulin secretion was observed in patients treated with the highest doses of liraglutide (1.25 mg and 1.9 mg). These doses led to a 118% and 103% increase in the first-phase insulin response, respectively. Interestingly, the second-phase insulin response was significantly increased only with the lower dose of the medication (1.25 mg) [108].

### 4.2. Pancreatitis Risk

Although a meta-analysis conducted in 2020 excluded the existence of an increased risk of pancreatic cancer and acute pancreatitis following the use of GLP-1RA [109], the consistently elevated levels of amylase and pancreatic lipase observed in many studies [110,111] still appear concerning. Additionally, one study reported a significant increase in amylase levels after patients received exenatide after a meal, although at the same time, that study excluded a significant increase in lipase levels [112].

In the LEADER Trial Investigators study, after 6 months of liraglutide treatment, lipase levels in a population with type 2 diabetes at high cardiovascular risk increased by 28%, and amylase levels increased by 7% from baseline [113]. However, the authors suggest that since pancreatitis was less common in patients receiving liraglutide, elevated levels of serum amylase and lipase were not predictive of subsequent acute pancreatitis events [113].

In a long-term evaluation (20 months), approximately a year after initiating exenatide therapy, 48 domestic cases (FDA’s Adverse Event Reporting System, U.S.) of acute pancreatitis were recorded from the date of the drug’s approval through the end of the year 2006 (though 18 patients were excluded from the statistics due to additional risk factors such as hypertriglyceridemia and alcohol abuse) [114]. In 2013, this study was repeated on a properly matched clinical and control group. This study took into account other risk factors for acute pancreatitis, such as alcohol consumption or gallstones. Ultimately, this paper indicated an increased risk of hospitalization due to acute pancreatitis in patients receiving exenatide [115]. Also, a meta-analysis made by Hong Ma et al. considering 61 randomized controlled trials showed that GLP-1RA administration poses a high risk of adverse events, with the most frequent serious adverse event being pancreatitis. Due to adverse events, higher odds of discontinuation had exenatide 10 µg, liraglutide 3.0 mg, and semaglutide 2.4 mg, but not dulaglutide 1.5 mg and semaglutide 1.0 mg [116]. Furthermore, a study on rats administered exenatide (5 µg/kg twice a day) continuously or intermittently for 10 weeks demonstrated chronic pancreatic damage in 30% of rats in the experimental group. This damage manifested as reduced size of acinar cells, infiltration of inflammatory cells, increased volume of cytoplasmic vacuoles, and elevated myelop–roxidase levels in the pancreas of rats [117].

In contrast to the aforementioned studies, many reports have indicated no increased risk of acute pancreatitis with GLP-1RA therapy [118,119,120,121,122]. A study conducted by the SUSTAIN-6 Investigators involving 1648 patients treated with semaglutide (using a fixed dose-escalation procedure from 0.25 mg to 0.5 mg over 4 weeks, up to a maintenance dose of 0.5 mg or 1.0 mg) showed that acute pancreatitis occurred in only 9 patients using semaglutide. In the placebo group, there were 12 such cases. All events were mild, but patients in the semaglutide group had elevated amylase levels [123]. Another clinical study conducted by the PIONEER 6 Investigators indicated that acute pancreatitis occurred in only one patient orally treated with semaglutide and in three patients receiving placebo [124]. A 2020 meta-analysis involving a group of 56,000 patients with type 2 diabetes confirmed the results of earlier studies, not indicating an increased risk of acute pancreatitis or pancreatic cancer after GLP-1RA therapy [109]. Also, a retrospective cohort study, including almost 350,000 patients with T2D, did not indicate a significant difference in the pancreatitis occurrence between patients treated with incretins vs. TZD. The incretin cohort had an incidence rate of 2.06 cases per 1000 patients, while for the TZD, the incidence rate was 1.94 cases per 1000 patients [125]. Furthermore, a study on mice fed a high-fat diet (16 weeks) and treated with semaglutide for 4 subsequent weeks (subcutaneous 40 μg/kg every three days) showed a great improvement of pancreatic architecture in tested animals. An improved turnover of islet heparan sulfate proteoglycans, hyaluronan, chondroitin sulfate proteoglycans, and collagens observed in this study possibly contribute to restoring a healthy islet functional milieu and decrease the formation of amyloid deposits [126], lowering the risk of T2D development [127].

However, a case report published in 2023, taking into account previous reports [128,129], discusses the possibility of dulaglutide-induced acute pancreatitis occurring in a long-term (two years) treated patient two weeks after increasing the dose of the drug [129]. This should alert healthcare professionals to the potential adverse effects of GLP-1RA use and provide more frequent follow-ups after changing the regimen of treatment.

### 4.3. GLP-1RA and Pancreatic Cancer

Scientific studies that compiled data from 43 trials comparing the effects of GLP-1RA (exenatide, liraglutide, lixisenatide, albiglutide, dulaglutide, or semaglutide) demonstrated no clear evidence of risk for pancreatitis, while data on pancreatic cancer are too scarce to draw any definite conclusions [130]. A meta-analysis involving clinical studies conducted on 56,004 patients with type 2 Diabetes Mellitus (T2DM) over a span of 1.3 to 5.4 years excluded the existence of an increased risk of pancreatic cancer in individuals using GLP-1RA. Reports of 108 cases of pancreatic cancer did not significantly differ from cases observed in the placebo group [109]. Independent and extensive studies conducted by the FDA and EMA have also shown that there is no increased risk of pancreatic cancer during GLP-1RA therapy [131].

However, since pancreatitis is a known risk factor for pancreatic cancer [132], conducted studies indicate that this matter should not be taken lightly. Research by Elashoff et al. also suggests a significantly increased risk of pancreatic cancer during combined exenatide and sitagliptin therapy [98]. This should prompt further research in this area, particularly due to the recent introduction of these drugs to the market.

## 5. Discussion

The development of GLP-1RA is undoubtedly a significant milestone in treating type 2 diabetes and obesity, which are at the forefront of modern lifestyle diseases. The action of GLP-1RA leads to the same metabolic effects as binding to the receptors of their endogenous counterparts, including increased insulin secretion, decreased glucagon secretion, anorexigenic effects, and modulation of cellular metabolism at the gene and protein expression level [133]. Those actions lead to an improvement in insulin signaling, weight loss, and decreased cardiovascular risk. The treatment with GLP-1RA is, in the majority of cases, a long-term or even a life-long solution.

**Figure 4:**
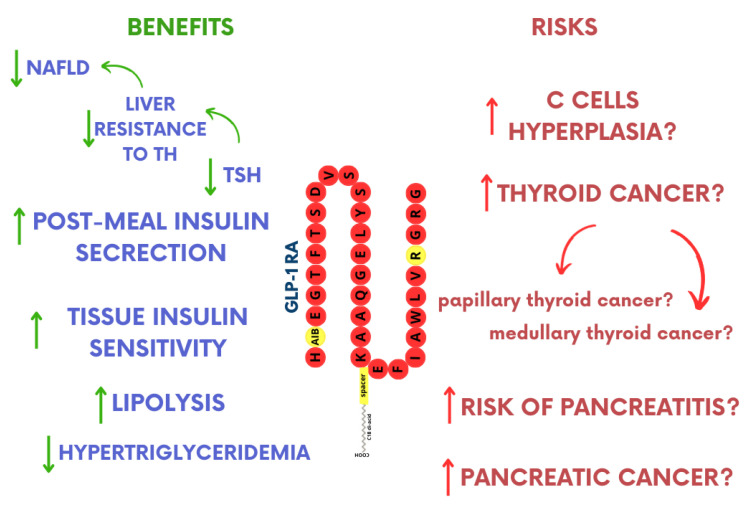
Schematic overview of the benefits and risks associated with GLP-1 receptor agonists. NFALD—non-alcoholic fatty liver disease; TH—thyroid hormones; TSH—thyroid-stimulating hormone; ↑- increased/increased risk; ↓- decreased/decreased risk.

This review summarized the current knowledge regarding the two most frequently mentioned potential side effects of GLP-1 RA use. While existing studies do not entirely rule out a link between the development of thyroid cancer (both medullary and papillary) and the use of GLP-1 RAs, clinical trials suggest no significant correlation between these occurrences in humans [88]. Particular caution should be exercised to patients at increased risk or diagnosed with medullary thyroid cancer or mutations in the RET proto-oncogene and multiple endocrine neoplasia type 2 (MEN2), as well as with a family history of the disease (Figure 4). Given the presence of GLP-1 receptors in various healthy and cancerous tissues, including the thyroid gland, caution should be advised when prescribing these medications. Patients eligible for GLP-1 RA therapy should undergo a thorough evaluation of thyroid function (including assessments of thyroid hormone levels and an ultrasound of the thyroid gland).

GLP-1 RAs undoubtedly exert a favorable effect on glucose metabolism [133]. However, people with diabetes constitute a unique group of patients, often with multiple comorbidities. Individuals with type 2 diabetes are often obese and experience chronic low-level inflammation [134]. This condition may further increase their risk of developing various diseases, including cancer [135]. The use of GLP-1 RAs in treating diabetes and obesity undeniably improves patients’ health and overall quality of life, both physically, mentally, and socially. It also reduces the risk of complications from these conditions, contributing to increased life expectancy and relieving pressure on the healthcare system. However, given the presence of chronic low-level inflammation, as well as the associated obesity, insulin resistance, hypertriacylglyceridemia, and its positive correlation with the development of pancreatitis [136], which is a risk factor for pancreatic cancer, patients in this group should exercise particular caution when using GLP-1 analogs and undergo regular monitoring.

## 6. Conclusions

Despite the favorable effects of GLP-1RA use on pancreatic endocrine function, reports suggest a risk of pancreatitis, particularly with prolonged use. Moreover, the risk of pancreatic cancer in GLP-1RA therapy has not been definitively refuted. In patients at increased risk of medullary thyroid cancer or mutations in the RET proto-oncogene and MEN2, as well as with a family history of thyroid diseases, prolonged use of GLP-1RA could contribute to the formation of thyroid tumors, particularly those originating from thyroid C-cells. In this review, we presented evidence that different patient subpopulations (e.g., patients with type 2 diabetes, non-alcoholic fatty liver disease, or those with/without thyroid disorders) respond differently to GLP-1RAs. As a result, the risk of developing acute inflammatory conditions or cancers may be higher in certain groups. Caution is, therefore, advised when prescribing these drugs to patients with multiple comorbidities. Physicians should also advise patients on the need for more frequent check-ups and encourage self-monitoring of their health. The use of GLP-1RAs requires continuous monitoring and further research.

With the increasing popularity of GLP-1RAs, to fully understand the influence of those drugs on the whole human organism and recognize the threat behind the GLP-1RA treatment, it is necessary to perform studies that primarily involve human tissues and cell cultures. Patient observation, adverse event reporting, and retrospective studies are required to understand all the effects and mechanisms of action of GLP-1RA and ensure patients’ safety.

## 7. Challenges, Limitations, and Future Scope

There are several challenges and limitations to this review. First and foremost, patients using GLP-1 analogs represent a heterogeneous population, which means that the effects of these drugs on their bodies are not uniform. This variability is not limited to coexisting conditions but also extends to different ethnic and age groups. These subpopulations exhibit different predispositions to various diseases, so the risk of developing pancreatitis or cancers may vary, particularly considering long-term exposure to GLP-1 RAs. Future studies should, therefore, focus on more narrowly defined patient groups and involve sufficiently large sample and control groups. Additionally, confounding factors that are risk factors for the diseases discussed in this review, such as underlying diabetes or obesity, should be accounted for in the statistical analysis of results.

Future research should also aim to better understand the mechanisms by which GLP-1 RAs might influence carcinogenesis, taking into account the differences in GLP-1 receptor expression in tissues between rodents and humans. Such studies should include in vivo analyses using human cell cultures to provide more relevant insights.

## Figures and Tables

**Table 1:** Approved glucagon-like peptide-1 analogs and receptor agonists by the Food and Drug Administration (FDA), China Food and Drug Administration (CFDA), and National Medical Products Administration (NMPA).

Name	Half-Life	Dosage	Approved Date
Exenatide	2.4 h	twice-daily injections	April 2005 (FDA)
Liraglutide	13 h	once-daily injections	January 2010 (FDA)
Exenatide	Exenatide extended-release, peak at 840 h [3]	once-weekly injections	January 2012 (FDA)
Albiglutide	120 h	once-weekly injections	April 2014 (FDA)
Dulaglutide	90 h	once-weekly injections	September 2014 (FDA)
Lixisenatide	3–4 h	once-daily injections	July 2016 (FDA)
Beinaglutide	1–2 min	three times-daily injections	December 2016 (NMPA)
Semaglutide	160 h	once-weekly injections	December 2017 (FDA)
	7 days	once-daily oral	September 2019
PEG-loxenatide	80 h	once-weekly injections	May 2019 (CFDA)
Tirzepatide	5 days	once-weekly injections	May 2022 (CFDA)

## Data Availability

No new data were created or analyzed in this study.

## List of Abbreviations

ACTH	Adrenocorticotropic hormone
Aib-2	Aminoisobutyric acid
cAMP	Cyclic adenosine monophosphate
CCH	C-cell hyperplasia
CFDA	China Food and Drug Administration
DPP-4	Dipeptidyl peptidase-4
EMA	European Medicines Agency
FAERS	FDA Adverse Event Reporting System
FDA	Food and Drug Administration
FT3	Triiodothyronine
FT4	Thyroxine
GLP-1RA	Glucagon-like peptide 1 receptor agonists
GLP-2	Glucagon-like peptide-2
GRPP	Glicentin-related pancreatic polypeptide
HIF-1	Hypoxia-inducible factor 1- α
HOMA-B	Homeostasis model assessment index of β-cel
IP-2	Intervening peptide-2
MEN 2	Multiple endocrine neoplasia type 2
NAFLD	Non-alcoholic fatty liver disease
NMPA	National Medical Products Administration
PC 1/3	Prohormone convertase 1/3
PEG	Polyethylene glycol
PTC	Papillary thyroid carcinoma
SCD	Stearoyl-CoA desaturase
SGLT2	Sodium-glucose cotransporter-2
T2DM	Type 2 diabetes mellitus
TH	Thyroid hormones
TSH	Thyroid-stimulating hormone
TZD	Thiazolidinediones
